# Bis{1-[(benzo­yloxy)meth­yl]-1*H*-1,2,3-benzotriazole-κ*N* ^3^}(nitrato-κ^2^ *O*,*O*′)­silver(I)

**DOI:** 10.1107/S1600536812006368

**Published:** 2012-03-03

**Authors:** Sen Xu, Yingzhong Shen

**Affiliations:** a Department of Applied Chemistry, School of Materials Science and Engineering, Nanjing University of Aeronautics and Astronautics, Nanjing, Jiangsu Province 210016, People’s Republic of China

## Abstract

In the crystal structure of the title coordination compound, [Ag(NO_3_)(C_14_H_11_N_3_O_2_)_2_], the Ag^I^ atom is four-coordinated in a distorted tetra­hedral geometry by two O atoms from one nitrate group and two N atoms from two different 1-[(benzo­yloxy)meth­yl]-1*H*-1,2,3-triazole ligands. In the complex, the two coordinated benzotriazole rings rings are nearly perpendicular, the dihedral angle between their planes being 87.08 (6)°.

## Related literature

For related structures, see: Han *et al.* (2008 ▶); Zhou *et al.* (2011 ▶).
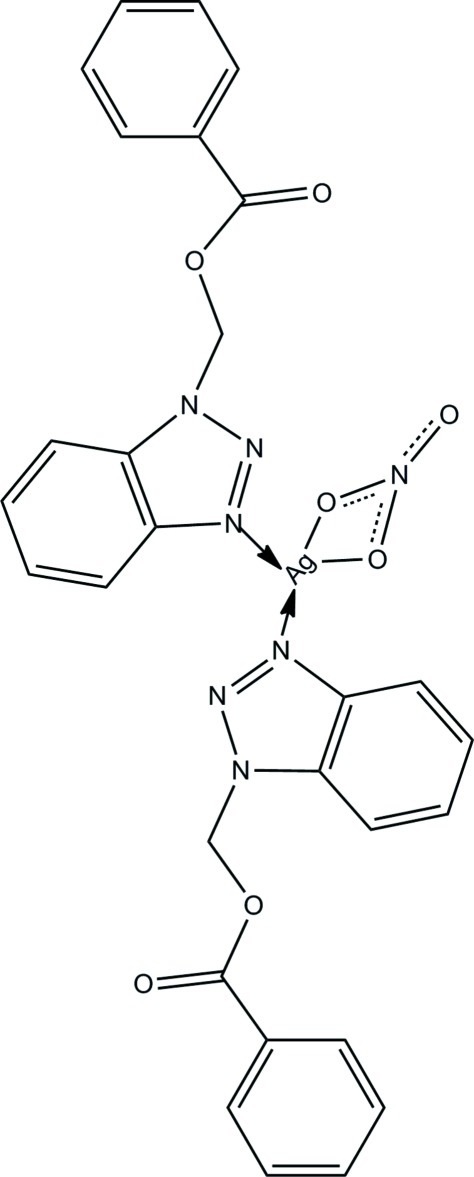


## Experimental

### 

#### Crystal data

[Ag(NO_3_)(C_14_H_11_N_3_O_2_)_2_]
*M* *_r_* = 676.40
Triclinic, 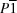

*a* = 9.8815 (5) Å
*b* = 10.6695 (5) Å
*c* = 15.0158 (7) Å
α = 70.405 (2)°
β = 73.323 (2)°
γ = 74.974 (2)°
*V* = 1405.21 (12) Å^3^
*Z* = 2
Mo *K*α radiation
μ = 0.78 mm^−1^
*T* = 296 K
0.20 × 0.18 × 0.17 mm

#### Data collection

Bruker SMART CCD area-detector diffractometer
Absorption correction: multi-scan (*SABADS*; Sheldrick, 1996 ▶) *T*_min_ = 0.860, *T*_max_ = 0.879
19832 measured reflections
4947 independent reflections
4563 reflections with *I* > 2σ(*I*)
*R*_int_ = 0.031

#### Refinement

*R*[*F* ^2^ > 2σ(*F* ^2^)] = 0.032
*wR*(*F* ^2^) = 0.067
*S* = 1.13
4947 reflections
404 parameters
1 restraint
H atoms treated by a mixture of independent and constrained refinement
Δρ_max_ = 0.31 e Å^−3^
Δρ_min_ = −0.46 e Å^−3^

### 

Data collection: *SMART* (Bruker, 2007 ▶); cell refinement: *SAINT* (Bruker, 2007 ▶); data reduction: *SAINT*; program(s) used to solve structure: *SHELXS97* (Sheldrick, 2008 ▶); program(s) used to refine structure: *SHELXL97* (Sheldrick, 2008 ▶); molecular graphics: *DIAMOND* (Brandenburg, 1999 ▶); software used to prepare material for publication: *SHELXTL* (Sheldrick, 2008 ▶).

## Supplementary Material

Crystal structure: contains datablock(s) global, I. DOI: 10.1107/S1600536812006368/vn2026sup1.cif

Structure factors: contains datablock(s) I. DOI: 10.1107/S1600536812006368/vn2026Isup2.hkl

Additional supplementary materials:  crystallographic information; 3D view; checkCIF report

## Figures and Tables

**Table 1:** Selected bond lengths (Å)

Ag1—N3	2.238 (2)
Ag1—N4	2.219 (2)
Ag1—O1	2.690 (2)
Ag1—O2	2.513 (2)

**Fig. 1.**
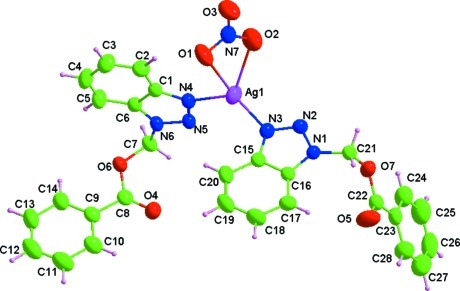
The molecular structure of the title compound with displacement ellipsoids drawn at the 50% probability level. Hydrogen atoms are drawn as spheres of arbitrary radius.

## supplementary crystallographic information

### Comment

Benzotriazol derivatives have been widely used for constructing complexes with transition metals (see *e.g.*: Han *et al.*, 2008; Zhou *et al.*, 2011). In this contribution, a new coordination compound was synthesized using (1*H*-benzo[*d*][1,2,3]triazol-1-yl)methyl benzoate and silver nitrate and characterized by single-crystal X-ray diffraction. The crystal structure of the title compound is shown in Fig.1. The Ag^I^ atom is four-coordinated in a slightly distorted tetradral geometry by two O atoms from one nitrate and two N atoms from two (1*H*-benzo[*d*][1,2,3]triazol-1-yl)methyl benzoate ligands. The bond distances Ag—N (Ag1—N3=2.238 (2) Å, Ag1—N4=2.219 (2) Å), and Ag—O (Ag1—O1=2.690 (2) Å, Ag1—O2=2.513 (2) Å) are within normal ranges.

### Experimental

(1*H*-benzo[*d*][1,2,3]triazol-1-yl)methyl benzoate (0.25 mmol) and silver nitrate (0.25 mmol) were mixed in a round bottom flask with 10 ml absolute ethyl alcohol, and stirred for 10 hours. After the completion of the reaction a white solid was obtained from which a small amount was dissolved in absolute ethyl alcohol, and single crystals were obtained by slow evaporation.

### Refinement

All H atoms were situated at idealized positions with carrier atom—H distances at 0.93Å for aryl groups, and they were treated using a riding model approximation with *U*_iso_(H) = 1.2*U*_eq_(C). H atoms bonded to methylene were refined independently with isotropic displacement parameters. The Ag1 and O2 sites were treated with a *SHELXL* DELU instruction.

### Crystal data

**Table:**

[Ag(NO_3_)(C_14_H_11_N_3_O_2_)_2_]	*Z* = 2
*M**_r_* = 676.40	*F*(000) = 684
Triclinic, *P*1	*D*_x_ = 1.599 Mg m^−^^3^
Hall symbol: -P 1	Mo *K*α radiation, λ = 0.71073 Å
*a* = 9.8815 (5) Å	Cell parameters from 9998 reflections
*b* = 10.6695 (5) Å	θ = 2.7–27.7°
*c* = 15.0158 (7) Å	µ = 0.78 mm^−^^1^
α = 70.405 (2)°	*T* = 296 K
β = 73.323 (2)°	Block, colourless
γ = 74.974 (2)°	0.20 × 0.18 × 0.17 mm
*V* = 1405.21 (12) Å^3^	

### Data collection

**Table:**

Bruker SMART CCD area-detector diffractometer	4947 independent reflections
Radiation source: fine-focus sealed tube	4563 reflections with *I* > 2σ(*I*)
Graphite monochromator	*R*_int_ = 0.031
φ and ω scans	θ_max_ = 25.0°, θ_min_ = 2.1°
Absorption correction: multi-scan (*SABADS*; Sheldrick, 1996)	*h* = −11→11
*T*_min_ = 0.860, *T*_max_ = 0.879	*k* = −12→12
19832 measured reflections	*l* = −17→17

### Refinement

**Table:**

Refinement on *F*^2^	Primary atom site location: structure-invariant direct methods
Least-squares matrix: full	Secondary atom site location: difference Fourier map
*R*[*F*^2^ > 2σ(*F*^2^)] = 0.032	Hydrogen site location: inferred from neighbouring sites
*wR*(*F*^2^) = 0.067	H atoms treated by a mixture of independent and constrained refinement
*S* = 1.13	*w* = 1/[σ^2^(*F*_o_^2^) + (0.0116*P*)^2^ + 0.9774*P*] where *P* = (*F*_o_^2^ + 2*F*_c_^2^)/3
4947 reflections	(Δ/σ)_max_ = 0.001
404 parameters	Δρ_max_ = 0.31 e Å^−^^3^
1 restraint	Δρ_min_ = −0.46 e Å^−^^3^

### Special details

**Table:**

Geometry. All e.s.d.'s (except the e.s.d. in the dihedral angle between two l.s. planes) are estimated using the full covariance matrix. The cell e.s.d.'s are taken into account individually in the estimation of e.s.d.'s in distances, angles and torsion angles; correlations between e.s.d.'s in cell parameters are only used when they are defined by crystal symmetry. An approximate (isotropic) treatment of cell e.s.d.'s is used for estimating e.s.d.'s involving l.s. planes.
Refinement. Refinement of *F*^2^ against ALL reflections. The weighted *R*-factor *wR* and goodness of fit *S* are based on *F*^2^, conventional *R*-factors *R* are based on *F*, with *F* set to zero for negative *F*^2^. The threshold expression of *F*^2^ > σ(*F*^2^) is used only for calculating *R*-factors(gt) *etc*. and is not relevant to the choice of reflections for refinement. *R*-factors based on *F*^2^ are statistically about twice as large as those based on *F*, and *R*- factors based on ALL data will be even larger.

### Fractional atomic coordinates and isotropic or equivalent isotropic displacement parameters (Å^2^)

**Table:**

	*x*	*y*	*z*	*U*_iso_*/*U*_eq_	
Ag1	0.03724 (2)	0.57849 (2)	0.088297 (16)	0.05262 (8)	
C1	−0.0863 (3)	0.3081 (2)	0.23322 (18)	0.0368 (5)	
C2	−0.1554 (3)	0.3546 (3)	0.3143 (2)	0.0483 (7)	
H2	−0.1642	0.4442	0.3128	0.058*	
C3	−0.2095 (3)	0.2608 (3)	0.3964 (2)	0.0568 (8)	
H3	−0.2560	0.2877	0.4522	0.068*	
C4	−0.1969 (3)	0.1258 (3)	0.3991 (2)	0.0580 (8)	
H4	−0.2344	0.0658	0.4568	0.070*	
C5	−0.1318 (3)	0.0794 (3)	0.32016 (19)	0.0498 (7)	
H5	−0.1249	−0.0100	0.3220	0.060*	
C6	−0.0759 (3)	0.1743 (2)	0.23637 (18)	0.0366 (5)	
C7	0.0367 (3)	0.0503 (3)	0.1079 (2)	0.0426 (6)	
C8	0.2437 (3)	−0.0951 (2)	0.16086 (19)	0.0395 (6)	
C9	0.2897 (3)	−0.2079 (3)	0.24241 (19)	0.0421 (6)	
C10	0.4303 (3)	−0.2301 (3)	0.2521 (2)	0.0560 (8)	
H10	0.4928	−0.1740	0.2085	0.067*	
C11	0.4774 (4)	−0.3351 (3)	0.3264 (3)	0.0682 (9)	
H11	0.5709	−0.3485	0.3336	0.082*	
C12	0.3867 (4)	−0.4196 (3)	0.3893 (2)	0.0690 (10)	
H12	0.4191	−0.4911	0.4387	0.083*	
C13	0.2474 (4)	−0.3988 (3)	0.3796 (2)	0.0666 (9)	
H13	0.1865	−0.4570	0.4224	0.080*	
C14	0.1973 (3)	−0.2923 (3)	0.3070 (2)	0.0544 (7)	
H14	0.1027	−0.2776	0.3015	0.065*	
C15	0.3679 (3)	0.6112 (2)	0.06858 (18)	0.0373 (6)	
C16	0.4652 (3)	0.6949 (2)	0.01166 (17)	0.0351 (5)	
C17	0.6031 (3)	0.6754 (3)	0.0275 (2)	0.0469 (7)	
H17	0.6678	0.7326	−0.0104	0.056*	
C18	0.6355 (3)	0.5662 (3)	0.1026 (2)	0.0567 (8)	
H18	0.7259	0.5482	0.1161	0.068*	
C19	0.5382 (4)	0.4799 (3)	0.1604 (2)	0.0557 (8)	
H19	0.5666	0.4066	0.2103	0.067*	
C20	0.4033 (3)	0.5004 (3)	0.1457 (2)	0.0483 (7)	
H20	0.3384	0.4440	0.1848	0.058*	
C21	0.4355 (3)	0.9080 (3)	−0.1274 (2)	0.0441 (6)	
C22	0.6299 (3)	0.8467 (3)	−0.2501 (2)	0.0446 (6)	
C23	0.6691 (3)	0.8206 (3)	−0.34636 (19)	0.0462 (7)	
C24	0.5683 (4)	0.8326 (3)	−0.3974 (2)	0.0638 (9)	
H24	0.4712	0.8594	−0.3725	0.077*	
C25	0.6129 (5)	0.8043 (4)	−0.4860 (3)	0.0789 (11)	
H25	0.5456	0.8125	−0.5210	0.095*	
C26	0.7561 (5)	0.7642 (4)	−0.5223 (2)	0.0769 (11)	
H26	0.7854	0.7444	−0.5815	0.092*	
C27	0.8553 (5)	0.7534 (4)	−0.4723 (3)	0.0802 (11)	
H27	0.9523	0.7267	−0.4974	0.096*	
C28	0.8126 (4)	0.7820 (4)	−0.3842 (2)	0.0660 (9)	
H28	0.8808	0.7752	−0.3504	0.079*	
H1A	0.356 (3)	0.977 (3)	−0.1326 (17)	0.037 (7)*	
H2A	0.510 (3)	0.935 (3)	−0.1106 (19)	0.047 (8)*	
H3A	−0.054 (3)	0.029 (3)	0.1051 (18)	0.041 (7)*	
H4A	0.102 (3)	0.072 (3)	0.048 (2)	0.045 (8)*	
N1	0.3925 (2)	0.7905 (2)	−0.05326 (14)	0.0363 (5)	
N2	0.2574 (2)	0.7682 (2)	−0.03701 (16)	0.0408 (5)	
N3	0.2419 (2)	0.6601 (2)	0.03592 (15)	0.0413 (5)	
N4	−0.0186 (2)	0.3735 (2)	0.14134 (15)	0.0385 (5)	
N5	0.0324 (2)	0.2868 (2)	0.09009 (14)	0.0374 (5)	
N6	−0.0017 (2)	0.1661 (2)	0.14623 (14)	0.0357 (5)	
N7	−0.1525 (3)	0.7667 (2)	0.20292 (16)	0.0459 (5)	
O1	−0.0939 (3)	0.6566 (2)	0.24968 (17)	0.0739 (7)	
O2	−0.1332 (3)	0.7897 (2)	0.11432 (15)	0.0781 (7)	
O3	−0.2257 (3)	0.8530 (2)	0.24265 (16)	0.0714 (7)	
O4	0.3217 (2)	−0.0385 (2)	0.08958 (14)	0.0524 (5)	
O5	0.7136 (2)	0.8368 (3)	−0.20369 (16)	0.0681 (6)	
O6	0.09795 (19)	−0.06300 (17)	0.17626 (13)	0.0451 (4)	
O7	0.48595 (19)	0.8807 (2)	−0.21973 (13)	0.0483 (5)	

### Atomic displacement parameters (Å^2^)

**Table:**

	*U*^11^	*U*^22^	*U*^33^	*U*^12^	*U*^13^	*U*^23^
Ag1	0.05344 (14)	0.04608 (14)	0.05684 (15)	−0.02381 (11)	0.00015 (10)	−0.01177 (10)
C1	0.0334 (13)	0.0346 (13)	0.0404 (13)	−0.0081 (11)	−0.0092 (11)	−0.0056 (11)
C2	0.0501 (16)	0.0422 (15)	0.0543 (17)	−0.0074 (13)	−0.0074 (13)	−0.0199 (13)
C3	0.0606 (19)	0.062 (2)	0.0452 (16)	−0.0159 (16)	0.0030 (14)	−0.0198 (15)
C4	0.068 (2)	0.0533 (18)	0.0432 (16)	−0.0226 (16)	0.0033 (14)	−0.0060 (14)
C5	0.0619 (18)	0.0394 (15)	0.0448 (15)	−0.0181 (14)	−0.0049 (14)	−0.0067 (12)
C6	0.0354 (13)	0.0365 (14)	0.0379 (13)	−0.0088 (11)	−0.0095 (11)	−0.0075 (11)
C7	0.0488 (17)	0.0329 (14)	0.0429 (15)	−0.0027 (12)	−0.0124 (13)	−0.0083 (12)
C8	0.0449 (15)	0.0325 (13)	0.0442 (15)	−0.0059 (12)	−0.0091 (12)	−0.0161 (11)
C9	0.0491 (16)	0.0340 (14)	0.0447 (15)	−0.0022 (12)	−0.0129 (12)	−0.0147 (11)
C10	0.0554 (18)	0.0443 (17)	0.070 (2)	−0.0069 (14)	−0.0224 (16)	−0.0125 (15)
C11	0.068 (2)	0.057 (2)	0.084 (2)	0.0001 (17)	−0.0385 (19)	−0.0165 (18)
C12	0.090 (3)	0.053 (2)	0.059 (2)	0.0075 (19)	−0.0331 (19)	−0.0116 (16)
C13	0.079 (2)	0.0532 (19)	0.0505 (18)	−0.0085 (17)	−0.0120 (17)	0.0020 (15)
C14	0.0580 (18)	0.0485 (17)	0.0489 (16)	−0.0069 (14)	−0.0115 (14)	−0.0061 (14)
C15	0.0380 (14)	0.0325 (13)	0.0404 (14)	−0.0050 (11)	−0.0066 (11)	−0.0117 (11)
C16	0.0364 (13)	0.0337 (13)	0.0341 (13)	−0.0031 (11)	−0.0072 (10)	−0.0111 (10)
C17	0.0364 (14)	0.0519 (17)	0.0514 (16)	−0.0054 (12)	−0.0105 (12)	−0.0146 (13)
C18	0.0496 (17)	0.0618 (19)	0.0608 (19)	−0.0003 (15)	−0.0267 (15)	−0.0150 (16)
C19	0.070 (2)	0.0445 (17)	0.0494 (17)	0.0031 (15)	−0.0252 (15)	−0.0085 (14)
C20	0.0600 (18)	0.0347 (14)	0.0448 (15)	−0.0086 (13)	−0.0081 (13)	−0.0069 (12)
C21	0.0386 (15)	0.0361 (15)	0.0483 (16)	−0.0064 (13)	−0.0051 (13)	−0.0043 (12)
C22	0.0413 (15)	0.0431 (15)	0.0450 (15)	−0.0094 (12)	−0.0100 (13)	−0.0051 (12)
C23	0.0511 (17)	0.0435 (15)	0.0378 (14)	−0.0125 (13)	−0.0101 (12)	−0.0003 (12)
C24	0.065 (2)	0.076 (2)	0.0505 (18)	−0.0199 (18)	−0.0161 (16)	−0.0107 (16)
C25	0.104 (3)	0.089 (3)	0.054 (2)	−0.032 (2)	−0.029 (2)	−0.0114 (19)
C26	0.122 (4)	0.064 (2)	0.0398 (17)	−0.028 (2)	−0.006 (2)	−0.0104 (16)
C27	0.087 (3)	0.087 (3)	0.053 (2)	−0.010 (2)	0.003 (2)	−0.0227 (19)
C28	0.058 (2)	0.081 (2)	0.0524 (18)	−0.0049 (18)	−0.0115 (16)	−0.0174 (17)
N1	0.0310 (11)	0.0339 (11)	0.0402 (11)	−0.0085 (9)	−0.0055 (9)	−0.0058 (9)
N2	0.0325 (11)	0.0382 (12)	0.0486 (13)	−0.0078 (9)	−0.0095 (10)	−0.0070 (10)
N3	0.0381 (12)	0.0376 (12)	0.0467 (12)	−0.0120 (10)	−0.0071 (10)	−0.0079 (10)
N4	0.0384 (12)	0.0321 (11)	0.0425 (12)	−0.0091 (9)	−0.0098 (9)	−0.0046 (9)
N5	0.0387 (12)	0.0328 (11)	0.0380 (11)	−0.0083 (9)	−0.0102 (9)	−0.0038 (9)
N6	0.0391 (11)	0.0308 (11)	0.0355 (11)	−0.0080 (9)	−0.0105 (9)	−0.0040 (9)
N7	0.0522 (14)	0.0410 (13)	0.0445 (13)	−0.0126 (11)	−0.0101 (11)	−0.0095 (11)
O1	0.0951 (18)	0.0394 (12)	0.0766 (15)	−0.0012 (12)	−0.0284 (14)	−0.0034 (11)
O2	0.107 (2)	0.0733 (14)	0.0446 (12)	0.0052 (11)	−0.0174 (13)	−0.0211 (11)
O3	0.0898 (17)	0.0583 (14)	0.0629 (14)	0.0131 (12)	−0.0205 (12)	−0.0298 (12)
O4	0.0490 (11)	0.0503 (12)	0.0503 (11)	−0.0117 (9)	−0.0046 (9)	−0.0078 (9)
O5	0.0436 (12)	0.1073 (19)	0.0633 (13)	−0.0018 (12)	−0.0169 (11)	−0.0413 (13)
O6	0.0439 (11)	0.0348 (10)	0.0467 (10)	−0.0026 (8)	−0.0100 (8)	−0.0023 (8)
O7	0.0395 (10)	0.0584 (12)	0.0406 (10)	−0.0123 (9)	−0.0082 (8)	−0.0037 (9)

### Geometric parameters (Å, º)

**Table:**

Ag1—N3	2.238 (2)	C15—C20	1.400 (4)
Ag1—N4	2.219 (2)	C16—N1	1.364 (3)
Ag1—O1	2.690 (2)	C16—C17	1.401 (4)
Ag1—O2	2.513 (2)	C17—C18	1.366 (4)
C1—N4	1.380 (3)	C17—H17	0.9300
C1—C6	1.391 (3)	C18—C19	1.403 (4)
C1—C2	1.395 (4)	C18—H18	0.9300
C2—C3	1.371 (4)	C19—C20	1.364 (4)
C2—H2	0.9300	C19—H19	0.9300
C3—C4	1.402 (4)	C20—H20	0.9300
C3—H3	0.9300	C21—O7	1.433 (3)
C4—C5	1.362 (4)	C21—N1	1.435 (3)
C4—H4	0.9300	C21—H1A	0.93 (3)
C5—C6	1.396 (3)	C21—H2A	0.98 (3)
C5—H5	0.9300	C22—O5	1.191 (3)
C6—N6	1.361 (3)	C22—O7	1.354 (3)
C7—O6	1.423 (3)	C22—C23	1.486 (4)
C7—N6	1.452 (3)	C23—C28	1.377 (4)
C7—H3A	1.00 (3)	C23—C24	1.379 (4)
C7—H4A	0.94 (3)	C24—C25	1.386 (5)
C8—O4	1.200 (3)	C24—H24	0.9300
C8—O6	1.361 (3)	C25—C26	1.372 (5)
C8—C9	1.484 (4)	C25—H25	0.9300
C9—C14	1.385 (4)	C26—C27	1.359 (5)
C9—C10	1.388 (4)	C26—H26	0.9300
C10—C11	1.381 (4)	C27—C28	1.379 (5)
C10—H10	0.9300	C27—H27	0.9300
C11—C12	1.367 (5)	C28—H28	0.9300
C11—H11	0.9300	N1—N2	1.355 (3)
C12—C13	1.379 (5)	N2—N3	1.304 (3)
C12—H12	0.9300	N4—N5	1.310 (3)
C13—C14	1.383 (4)	N5—N6	1.348 (3)
C13—H13	0.9300	N7—O3	1.226 (3)
C14—H14	0.9300	N7—O2	1.235 (3)
C15—N3	1.381 (3)	N7—O1	1.239 (3)
C15—C16	1.384 (3)		
			
N4—Ag1—N3	135.16 (8)	C18—C17—H17	122.5
N4—Ag1—O2	124.85 (8)	C16—C17—H17	122.5
N3—Ag1—O2	98.41 (8)	C17—C18—C19	122.9 (3)
N3—Ag1—O1	104.13 (8)	C17—C18—H18	118.6
N4—Ag1—O1	97.67 (7)	C19—C18—H18	118.6
O1—Ag1—O2	48.14 (7)	C20—C19—C18	122.0 (3)
N4—C1—C6	107.6 (2)	C20—C19—H19	119.0
N4—C1—C2	131.2 (2)	C18—C19—H19	119.0
C6—C1—C2	121.1 (2)	C19—C20—C15	116.3 (3)
C3—C2—C1	116.3 (3)	C19—C20—H20	121.9
C3—C2—H2	121.9	C15—C20—H20	121.9
C1—C2—H2	121.9	O7—C21—N1	110.4 (2)
C2—C3—C4	122.1 (3)	O7—C21—H1A	106.5 (15)
C2—C3—H3	119.0	N1—C21—H1A	109.1 (15)
C4—C3—H3	119.0	O7—C21—H2A	110.9 (16)
C5—C4—C3	122.3 (3)	N1—C21—H2A	109.2 (16)
C5—C4—H4	118.8	H1A—C21—H2A	111 (2)
C3—C4—H4	118.8	O5—C22—O7	123.4 (3)
C4—C5—C6	115.9 (3)	O5—C22—C23	124.7 (3)
C4—C5—H5	122.0	O7—C22—C23	111.9 (2)
C6—C5—H5	122.0	C28—C23—C24	119.8 (3)
N6—C6—C1	104.6 (2)	C28—C23—C22	117.5 (3)
N6—C6—C5	133.1 (2)	C24—C23—C22	122.7 (3)
C1—C6—C5	122.3 (2)	C23—C24—C25	119.4 (3)
O6—C7—N6	108.1 (2)	C23—C24—H24	120.3
O6—C7—H3A	106.2 (15)	C25—C24—H24	120.3
N6—C7—H3A	107.7 (15)	C26—C25—C24	120.1 (4)
O6—C7—H4A	111.9 (16)	C26—C25—H25	119.9
N6—C7—H4A	108.1 (17)	C24—C25—H25	119.9
H3A—C7—H4A	115 (2)	C27—C26—C25	120.3 (3)
O4—C8—O6	123.1 (2)	C27—C26—H26	119.8
O4—C8—C9	126.0 (2)	C25—C26—H26	119.8
O6—C8—C9	111.0 (2)	C26—C27—C28	120.1 (4)
C14—C9—C10	119.8 (3)	C26—C27—H27	119.9
C14—C9—C8	121.7 (3)	C28—C27—H27	119.9
C10—C9—C8	118.5 (3)	C23—C28—C27	120.2 (3)
C11—C10—C9	120.2 (3)	C23—C28—H28	119.9
C11—C10—H10	119.9	C27—C28—H28	119.9
C9—C10—H10	119.9	N2—N1—C16	110.71 (19)
C12—C11—C10	120.1 (3)	N2—N1—C21	119.2 (2)
C12—C11—H11	120.0	C16—N1—C21	129.9 (2)
C10—C11—H11	120.0	N3—N2—N1	107.9 (2)
C11—C12—C13	120.1 (3)	N2—N3—C15	109.0 (2)
C11—C12—H12	120.0	N2—N3—Ag1	121.42 (16)
C13—C12—H12	120.0	C15—N3—Ag1	129.61 (16)
C12—C13—C14	120.7 (3)	N5—N4—C1	108.88 (19)
C12—C13—H13	119.7	N5—N4—Ag1	121.83 (15)
C14—C13—H13	119.7	C1—N4—Ag1	128.42 (17)
C13—C14—C9	119.2 (3)	N4—N5—N6	108.24 (19)
C13—C14—H14	120.4	N5—N6—C6	110.6 (2)
C9—C14—H14	120.4	N5—N6—C7	120.9 (2)
N3—C15—C16	108.2 (2)	C6—N6—C7	128.4 (2)
N3—C15—C20	130.6 (2)	O3—N7—O2	120.0 (2)
C16—C15—C20	121.2 (2)	O3—N7—O1	121.2 (2)
N1—C16—C15	104.3 (2)	O2—N7—O1	118.7 (3)
N1—C16—C17	133.0 (2)	N7—O2—Ag1	100.98 (17)
C15—C16—C17	122.7 (2)	C8—O6—C7	117.8 (2)
C18—C17—C16	115.0 (3)	C22—O7—C21	116.8 (2)
			
N4—C1—C2—C3	−178.6 (3)	O7—C21—N1—C16	98.7 (3)
C6—C1—C2—C3	1.0 (4)	C16—N1—N2—N3	−0.6 (3)
C1—C2—C3—C4	−0.2 (5)	C21—N1—N2—N3	−177.1 (2)
C2—C3—C4—C5	−0.8 (5)	N1—N2—N3—C15	0.4 (3)
C3—C4—C5—C6	1.0 (5)	N1—N2—N3—Ag1	−178.42 (15)
N4—C1—C6—N6	0.3 (3)	C16—C15—N3—N2	−0.1 (3)
C2—C1—C6—N6	−179.3 (2)	C20—C15—N3—N2	178.3 (3)
N4—C1—C6—C5	178.9 (2)	C16—C15—N3—Ag1	178.64 (16)
C2—C1—C6—C5	−0.8 (4)	C20—C15—N3—Ag1	−2.9 (4)
C4—C5—C6—N6	177.9 (3)	N4—Ag1—N3—N2	140.36 (18)
C4—C5—C6—C1	−0.2 (4)	O2—Ag1—N3—N2	−54.18 (19)
O4—C8—C9—C14	−161.0 (3)	O1—Ag1—N3—N2	−103.01 (19)
O6—C8—C9—C14	18.4 (3)	N4—Ag1—N3—C15	−38.2 (3)
O4—C8—C9—C10	17.5 (4)	O2—Ag1—N3—C15	127.2 (2)
O6—C8—C9—C10	−163.0 (2)	O1—Ag1—N3—C15	78.4 (2)
C14—C9—C10—C11	−0.6 (4)	C6—C1—N4—N5	−0.6 (3)
C8—C9—C10—C11	−179.2 (3)	C2—C1—N4—N5	179.0 (3)
C9—C10—C11—C12	1.5 (5)	C6—C1—N4—Ag1	−169.87 (16)
C10—C11—C12—C13	−1.0 (5)	C2—C1—N4—Ag1	9.7 (4)
C11—C12—C13—C14	−0.4 (5)	N3—Ag1—N4—N5	−55.2 (2)
C12—C13—C14—C9	1.3 (5)	O2—Ag1—N4—N5	142.44 (17)
C10—C9—C14—C13	−0.8 (4)	O1—Ag1—N4—N5	−174.17 (18)
C8—C9—C14—C13	177.7 (3)	N3—Ag1—N4—C1	112.9 (2)
N3—C15—C16—N1	−0.3 (3)	O2—Ag1—N4—C1	−49.5 (2)
C20—C15—C16—N1	−178.9 (2)	O1—Ag1—N4—C1	−6.1 (2)
N3—C15—C16—C17	178.2 (2)	C1—N4—N5—N6	0.6 (3)
C20—C15—C16—C17	−0.4 (4)	Ag1—N4—N5—N6	170.76 (14)
N1—C16—C17—C18	178.9 (3)	N4—N5—N6—C6	−0.4 (3)
C15—C16—C17—C18	0.9 (4)	N4—N5—N6—C7	177.9 (2)
C16—C17—C18—C19	−0.4 (4)	C1—C6—N6—N5	0.1 (3)
C17—C18—C19—C20	−0.6 (5)	C5—C6—N6—N5	−178.3 (3)
C18—C19—C20—C15	1.1 (4)	C1—C6—N6—C7	−178.2 (2)
N3—C15—C20—C19	−178.8 (3)	C5—C6—N6—C7	3.5 (5)
C16—C15—C20—C19	−0.6 (4)	O6—C7—N6—N5	133.5 (2)
O5—C22—C23—C28	0.2 (5)	O6—C7—N6—C6	−48.4 (3)
O7—C22—C23—C28	−178.6 (3)	O3—N7—O1—Ag1	−176.1 (2)
O5—C22—C23—C24	179.6 (3)	O2—N7—O1—Ag1	2.3 (3)
O7—C22—C23—C24	0.9 (4)	N4—Ag1—O1—N7	−132.13 (17)
C28—C23—C24—C25	0.6 (5)	N3—Ag1—O1—N7	87.37 (17)
C22—C23—C24—C25	−178.9 (3)	O2—Ag1—O1—N7	−1.32 (16)
C23—C24—C25—C26	0.3 (5)	O3—N7—O2—Ag1	175.9 (2)
C24—C25—C26—C27	−0.7 (6)	O1—N7—O2—Ag1	−2.5 (3)
C25—C26—C27—C28	0.4 (6)	N4—Ag1—O2—N7	67.4 (2)
C24—C23—C28—C27	−1.0 (5)	N3—Ag1—O2—N7	−100.12 (19)
C22—C23—C28—C27	178.5 (3)	O1—Ag1—O2—N7	1.35 (16)
C26—C27—C28—C23	0.5 (6)	O4—C8—O6—C7	−5.3 (4)
C15—C16—N1—N2	0.6 (3)	C9—C8—O6—C7	175.3 (2)
C17—C16—N1—N2	−177.7 (3)	N6—C7—O6—C8	−97.7 (3)
C15—C16—N1—C21	176.5 (2)	O5—C22—O7—C21	1.2 (4)
C17—C16—N1—C21	−1.8 (5)	C23—C22—O7—C21	179.9 (2)
O7—C21—N1—N2	−85.7 (3)	N1—C21—O7—C22	−97.4 (3)
